# A Novel Load-Sharing System to Simulate the Creep of Strain-Hardening Cementitious Composites (SHCCs) in Practical Situations

**DOI:** 10.3390/ma17102407

**Published:** 2024-05-17

**Authors:** Karuna Arachchige Shan Dilruksha Ratnayake, Christopher Kin Ying Leung

**Affiliations:** Department of Civil and Environmental Engineering, Hong Kong University of Science and Technology, Hong Kong SAR, China; kasdr@connect.ust.hk

**Keywords:** creep, restrained, strain-hardening cementitious composite, engineered cementitious composite

## Abstract

The ductility and exhibition of the multiple, fine, self-controlled cracking of strain-hardening cementitious composites (SHCCs) under tension has made them attractive for enhancing the durability of civil infrastructure. These fine cracks are key to preventing the ingress of water and harmful chemicals into the structure and thereby achieving steel reinforcement. However, several studies have suggested that the short-term fine cracks shown in the laboratory may end up exceeding the acceptable crack widths that are specified in design codes when SHCC members are subjected to sustained constant loads. In real structures, however, the load is also shared by the steel reinforcement in the member, so the SHCC within may not be under a constant load; therefore, the crack widening will not be as severe. This study focuses on the creep behaviour of SHCCs when they are applied as an external layer on reinforced concrete to enhance durability. A novel approach to simulate various stress–strain regimes in such systems is developed by using a fixture to share a sustained moment exclusively between a reinforcement member and SHCC. The developed load-sharing system allows stresses within the reinforcement and SHCC to be monitored against time during the imposed loading, while ensuring access to the SHCC layer for instrumentation and monitoring of strain/cracking. The time-dependent widening of cracks in the SHCC layer is found to be much less significant than that under constant loading, so resistance to water/chemical penetration can still be ensured in the long term. The obtained information on the variation in stress, strain, and crack opening with time will be useful for the development of a general model for the creep behaviour of SHCC members.

## 1. Introduction

Strain-hardening cementitious composites (SHCCs) are high-performance, fibre-reinforced cementitious materials showing very high ductility [1,2]; typically, they employ polymeric short fibres distributed randomly in a cementitious matrix, where a specially engineered matrix–fibre interaction brings forth tensile strain-hardening behaviour. SHCCs are also referred to using alternate nomenclature: engineered cementitious composites (ECCs) or pseudo-ductile cementitious composites. The pseudo-ductility of SHCCs is derived from the formation of multiple small cracks across the element, as opposed to a single/few cracks which widen, as is the case for conventional reinforced concrete (RC) and fibre-reinforced cementitious materials [2,3,4,5]. Cracks in SHCCs typically remain below 100 µm, which is very favourable in terms of durability [6,7,8,9,10].

Such a tensile performance of SHCCs can be exploited when they are used together with concrete, with the goal of enhancing durability (as conventional concrete is weak in tensile performance and control of cracks). Specifically, the formation of cracks wider than 100 microns in concrete makes it highly susceptible to water/chemical ingress [6]. In the Hong Kong Code of Practice [11], the upper bound for crack width was established to be 300 μm, while a maximum of 200 μm was recommended for structures requiring watertightness (e.g., basement wall below the underground water table). Maalej and Li [12] showed that the ingress of aggressive substances can be slowed down by a significant margin with the use of SHCCs in the bottom third (on the tension side) of an RC flexural member. To serve a similar purpose, RC members can be made with an SHCC permanent formwork that can effectively control the surface crack width [13]. In the above approaches, SHCCs are only employed near the tensile surface so that a good performance/cost ratio can be achieved.

A majority of studies on SHCCs investigate aspects related to their short-term loading behaviour. The limited studies on their long-term behaviour have highlighted that it may not be conservative to assume a short-term performance for crack control when SHCCs are used in real structures, where stresses and strains are sustained over the lifetime of the structure. In a study carried out by Boshoff et al. [14], constant stresses over time induced the formation of new cracks given a sufficiently high load. In addition to this phenomenon, the width of a crack in samples preloaded to a 1% strain almost tripled from 78 µm to 244 µm in 8 months. The occurrence of creep failure at 40–50% of tensile strength [15] and the localisation of cracks in notched specimens within weeks [16] were also demonstrated by Boshoff et al.

In a creep test imposing a constant stress, the allowed deformation is unbound. Conversely, in a relaxation test, the deformation is kept constant, allowing the stress to change. Both are different from the stress–strain variations in actual structures, where the deformation can increase but is restricted by serviceability design criteria. In a situation with where SHCCs are applied as a layer on the tensile face of an RC member to improve durability, the used SHCC will share the loading with the concrete and steel reinforcement. The SHCC stress in this case most likely reduces with time because SHCCs show a higher creep rate than concrete and steel. The ensuing force redistribution results in load transfer from SHCC to other elements. As such, the cracking behaviour in a practical scenario may be significantly different to that exhibited when SHCC is subjected to constant stresses.

In view of the limitation of the constant-stress creep test, the authors have designed experiments to simulate the long-term mechanical behaviour of SHCCs under a more realistic situation. In [17], a reinforced concrete beam with SHCC strips cast on the surface was put under constant loading (at serviceability load level) for 6 months to observe the cracking behaviour. The crack widths in the SHCC were shown to be within the acceptable limits in the long term. To focus on the creep/relaxation behaviour of the SHCC layer, a new loading setup enabling load sharing between an SHCC layer and a rebar embedded inside two concrete half beams (connected with a hinge) was proposed and preliminary results were reported in a conference paper [18]. The crack width development was also found to be less significant than the case of constant stress on SHCC. In both the studies, however, aspects that are critical for the characterisation of SHCCs, such as the SHCC stress variation in the system, were not monitored. In the current study, the proposed experimental setup in [18] is further refined to simulate and monitor the creep behaviour of an SHCC layer placed on the surface of a flexural member in a more realistic manner to present a complete study on the novel testing method.

The proposed setup allows the sharing of a constant moment between the SHCC layer and a reinforcing bar. To provide a comprehensive understanding of the testing method, the design of the setup and testing procedures is described in detail in the following sections. After loading the SHCC layer to a certain strain level, the time-dependent development of strain and crack width was monitored. While the previous study [17,18] only focuses on crack development with strain, the derivation of SHCC stress based on the measurement of the strain (and stress) of the calibrated rebar is presented here for the first time. Together with strain measurement on the SHCC, the variation in both stress and strain with time during the creep test can thus be determined. By using different rebars to change the load redistribution, the feasibility of generating different stress vs. strain relations during the creep test was shown. In addition, a method for deriving the creep compliance of SHCC from the stress vs. strain relation is proposed. We believe that the results of this study can demonstrate the applicability of the setup in the study of tensile creep behaviour in SHCCs in practical situations.

## 2. Experimental Program

**Figure 1:**
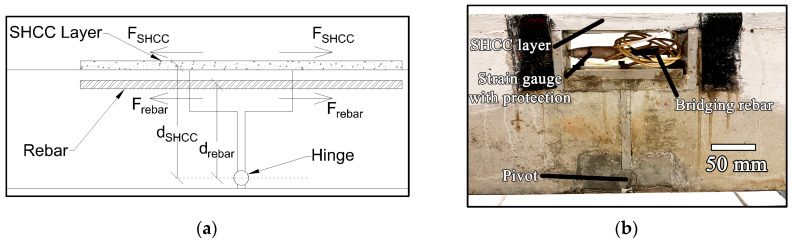
(**a**) Schematic illustration of an analogous tension load-sharing system between rebar and SHCC; (**b**) a photograph of the sharing system following the removal of SHCC formwork.

The characterisation of SHCC behaviour subjected to creep deformation scenarios that are likely to be found in practice formed the basis of the experiment. For example, SHCCs can be applied to the tension surface of an RC flexural member with the goal of enhancing durability. In the analytical approach for section analysis, the contribution from the tensile region of concrete is not considered in the moment equilibrium. Such a sharing of a constant moment between the rebar and an SHCC exclusively is facilitated in the arrangement shown in Figure 1a, where a constant moment is resisted in the vertical plane of the hinge. As the creeping rate of the SHCC is much faster than the bridging rebar creep, the stress in SHCC drops with time, while the rebar stress increases. The situation is physically similar to an RC beam or slab with a permanent formwork at its bottom. It can also reflect the behaviour of the beam studied by Maalej [12], with the lower part made with an SHCC. In that case, the thicker SHCC layer carries a larger portion of the loading, but the share of loading continues to decrease as creeping occurs. Indeed, even for a full SHCC beam reinforced by steel rebar, the relaxation of the SHCC is expected to occur under constant applied loading because creep deformation will increase the strain and hence the loading carried by the steel rebar. Qualitative observations and findings from the test can therefore be applied to more general cases. Derivation of creep compliance from the test results for the quantitative analysis of beam members will be discussed in a later section.

Considering the low initial strain values expected in real world situations (<2%), the proposed load sharing arrangement was implemented by using a frame consisting of two RC half beams bridged by a rebar, as shown in Figure 1b. It was tested under a 4-point bending arrangement with the loading points and supports, as indicated in the figure. The RC frame was 1.7 m long with a cross-section of 0.1 m × 0.2 m. A discontinuity of 175 mm at the centre of the beam (including the thickness of the PVC formwork) was created over a 70 mm height at the top, and a 12 mm discontinuity was created from thereon to the bottom. The 25 mm steel pin in contact with the groove (having the same radius as the steel pin) of a steel block allowed the rotation of the half beams about the pin in the vertical plane. Anchors connected to the steel block firmly fixed it within the RC half beam.

This restrained creep test setup acted as a fixture for testing the SHCC in a restrained condition. Medium-strength concrete placement followed the positioning of the rebar (with a strain gauge attached), connecting the half beams at 25 mm below the top surface of the RC half beams. The central 150 mm of the rebar (175 mm minus the total thickness of PVC formwork on the two sides) between the two half beams was exposed to air, and a length of 163 mm was embedded in each half beam. Subtracting the cover thickness, the lap length between the top reinforcement of the concrete beam and the bridging rebar was therefore 151 mm on each side.

Two types of bridging rebar were used in this study: 8 mm nominal Ø high-yield steel bar (having yield strength of ~500 MPa) and 6 mm nominal Ø glass-fibre-reinforced polymer (GFRP) bar (having a tensile strength of ~1700 MPa GPa). The higher elastic modulus of steel compared to GFRP allows for a faster relaxation of the SHCC layer. The concrete half beam on each side was reinforced by a single rebar of the same diameter and type as the bridging rebar, in tension. Two high-yield steel bars measuring 10 mm Ø were used for compression. Mild steel stirrups measuring 6 mm Ø were spaced at 125 mm as shear reinforcement. A cover of 12 mm was maintained on all sides.

**Figure 2:**
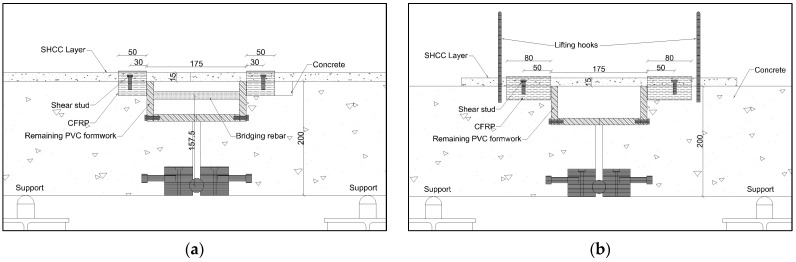
Schematic illustrations of the (**a**) the creep test setup and (**b**) quasistatic testing setup without bridging rebar.

To prevent slip at the SHCC–concrete interface close to the edges above the hinge, the concrete surface was roughened and two shear studs each were fixed on each side. Additionally, a carbon-fibre-reinforced polymer (CFRP) layer was pasted onto the SHCC surface at the ends of the 175 mm gauge length to prevent cracks forming beyond the gauge length. A schematic illustration describing the dimensions is provided in Figure 2a. A single linear variable differential transformer (LVDT) placed just outside the gauge length captured the deformation within.

The concrete used in this study had a compressive strength of 63 MPa and 70 MPa at 7 and 28 days, respectively. The polyethylene-based SHCC adopted from Chen et al. [19] consisted of 0.8:0.2:0.5 of cement/silica fume/silica sand mass ratio with a water binder ratio of 0.18 by mass and 2.2% polyethylene fibres by volume. An average tensile strain capacity of 2.8% and a compressive strength of 132 MPa at 14 days was shown by the SHCC.

### 2.1. Direct Tensile Behaviour of SHCC Layer Obtained from the Loading System

Theoretically, the setup would introduce slightly different strains on the lower and upper sides of the SHCC layer. It is of interest to check whether the loading-system-induced stress–strain behaviour in the SHCC layer is similar to that during a direct tension test performed on a conventional universal testing machine. This would ensure that the non-uniformity throughout the depth of the layer’s cross-section has little effect on the results.

To compare the similarity of the tensile stress–strain of the SHCC layer with the quasistatic direct tension test results, the setup was modified by using the fixture without the bridging rebar, as indicated in Figure 1a. With the absence of the bridging rebar, the total applied moment was counterbalanced only by the force generated in the SHCC layer. By loading the fixture (and thereby the SHCC layer to induce quasistatic failure), the tensile stress–strain relation of the SHCC was found. The stress on the SHCC was calculated by using the moment equilibrium around the pivot, and the strain was calculated using the LVDT readings. A rectangular-shaped 15 mm (thickness) × 100 mm (width) SHCC was laid over the central 500 mm of the setup was adopted for this test as shown in the schematic illustration in Figure 2b. Two shear studs were placed 50 mm from the inside edge, 30 mm from the longitudinal edges on either side; these were fixed as described in Section 2.3.

Two 10 mm Ø high-yield steel bars served as tension reinforcement and the same was adopted for compression reinforcement. Stirrups and concrete cover followed the same configuration as that mentioned in Section 2.

The setup with a rectangular-shaped SHCC layer would result in a linear strain field in the central region as opposed to a dog-bone-shaped layer, where the stress–strain field changed when the layer width varies along the length. The resulting stress–strain characteristics were then compared with those obtained from the direct tension tests performed using accompanying 15 mm rectangular (or coupon) specimens cast along with the SHCC layer.

### 2.2. Installation of Strain Gauge on Bridging Rebar and Pre-Calibration

Prior to placement of concrete, a single strain gauge was attached to the rebar at its central 150 mm region. The purpose was to monitor the strain (and stress) carried by the rebar at varying load levels. The stress in the SHCC can then be obtained from moment equilibrium about the pivot. To ensure accurate measurements in the long term, a special procedure for installing and protecting the strain gauge was followed.

To prevent distortions in strain gauge readings due to moisture migration [20] and adhesive embrittlement with age [21], a more resilient adhesive and protection suitable for long-term monitoring was adopted instead of using common cyanoacrylate adhesive to fix the strain gauges onto the rebar. The FLAB-10-11 (from TML™, Tokyo, Japan) strain gauge was bonded to the grinded rebar surface with epoxy (EB-2 by TML™). The thickness of the epoxy layer between the strain gauge and the rebar was reduced by applying even pressure on the strain gauge (to ensure minimal shear strain in the bonding material during future loading), followed by curing in ambient conditions for 24 h.

A layer of microcrystalline wax (W-1 by TML™) was then applied to a sufficient thickness, covering the strain gauge and terminal followed by the application of a butyl rubber (SB Tape by TML™) layer on the wax, making the first two protective coatings. A vinyl/mastic (VM Tape by 3M™, Maplewood, MN, USA) was wrapped around the location of the strain gauge, which was in turn bound by a self-bonding tape to secure the coating. A third coating was applied by wrapping a bandage impregnated with epoxy (P-2 from TML™). A more reliable bond is expected between the strain gauge and the rebar by fastening the strain gauge using epoxy, while the multilayer protection is expected to prevent deleterious substances from reaching the strain gauge–rebar bond interface.

After installing the strain gauge, the rebar was pre-calibrated in a universal testing machine (UTM) by attaching an extensometer over a length of the rebar, which was not grinded. The rebar was then loaded to 40% of yield stress and the extensometer strains ($Strain_{extensometer}$) were compared against the strain read by the strain gauge ($Strain_{strain\;gauge}$). The elastic modulus of the rebar was determined using the strains recorded by the extensometer. To paste the strain gauges, the diameter of the rebar was reduced slightly due to the grinding process. As the reduced area at the strain gauge location was difficult to measure directly, it was determined based on the gradients of the tensile stress–strain graph of the rebar (σ−ε Gradient), obtained from the strain gauge and extensometer. The possible effects of an imperfect parallel alignment of the strain gauge to the rebar were also captured within $A_{eff}$ with this calibration. Based on elasticity,

(1) $$\sigma -\varepsilon \;Gradient_{strain\;gauge}=\frac{Force/{Area}_{strain\;gauge}}{Strain_{strain\;gauge}}$$

where $Area_{strain\;gauge}$ refers to the area of the rebar at the location of the strain gauge

(2) $$\sigma -\varepsilon \;Gradient_{extensometer}=\frac{Force/{Area}_{extensometer}}{Strain_{extensometer}}$$

where ${Area}_{extensometer}$ refers to the area of the rebar at the location of the extensometer (i.e., the nominal area of the steel bar, as this location is not grinded).

Taking the ratio of Equations (1) and (2),

(3) $$\frac{\sigma -\varepsilon \;Gradient_{strain\;gauge}}{\sigma -\varepsilon \;Gradient_{extensometer}}=\frac{{Area}_{extensometer}\times Strain_{extensometer}}{{Area}_{strain\;gauge}\times Strain_{strain\;gauge}}$$

The ratio of the gradient in Equation (3) was used as the correction factor to compute the effective area of the rebar at the location of the strain gauge, from the nominal area. Once the effective area was obtained, the change in force in the rebar was determined from the elastic modulus and the change in measured strain.

### 2.3. SHCC Layer Placement

Shortly after the concrete half beams were cast, the aggregate at the top surface of the concrete in the centre span was exposed using a waterjet. This procedure served to roughen the surface and create a better SHCC/concrete bond when the SHCC was placed after the beam has been cured for 14 days.

Two shear studs were placed 30 mm from the inside edge and 30 mm from the longitudinal edges on either side. The studs were fixed by drilling 15 mm holes into the concrete and using epoxy to hold it in position, such that the stud protruded to 7 mm above the concrete surface.

A 15 mm thick, 100 mm wide layer of SHCC was then placed on the central 1400 mm of the top surface. During casting, a plywood formwork arrangement in the central 150 mm gap supported the SHCC from its bottom. The SHCC and concrete were then cured until 14 days after casting the SHCC. Epoxy was used to paste 50 mm long CFRP strips on the SHCC layer, extending down the side face of the concrete member.

### 2.4. Loading and Monitoring

**Figure 3:**
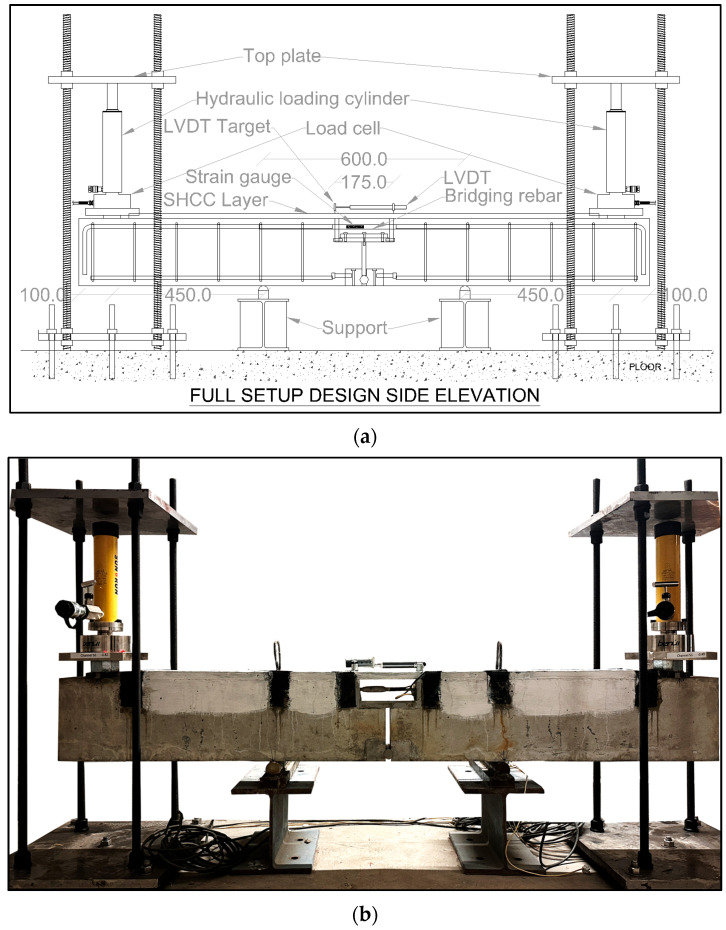
(**a**) Schematic illustration and (**b**) photograph of the loading system for the fixture.

The system was subjected to 4-point bending, resulting in a constant moment zone in the central 600 mm. RSC-106 hydraulic cylinders placed 100 mm from each end was used to load and maintain the specific load (with the aid of SV-82 valves). A load cell was attached to the bottom of the hydraulic cylinders, which in turn rested on a 50 mm steel strip glued to the top surface of the beam. The initial loading was controlled using a manual hydraulic pump. An illustration and photograph of the arrangement is shown in Figure 3. The steel pin was cleaned and lubricated with grease before loading. A thin layer of white paint was applied on the surface of the SHCC so cracks were easily discernible.

For the quasistatic tests of the SHCC layer alone, the load from the hydraulic cylinders on the two sides were increased, such that both were within 1 kN of each other (as the load at both ends should be equal to induce a constant moment within the midspan). The test continued until the SHCC failed.

For long-term testing, a constant load level of 4 kN and 6 kN was adopted for the GFRP and steel bridging rebar setup, respectively. In the absence of the SHCC layer (or in the event of complete relaxation the SHCC layer), these loads would correspond to a stress of 404 MPa and 341 MPa in the GFRP and steel rebar, respectively. With these levels of load, the initial strain for both cases were predicted to be similar, while the strain increase with the drop in SHCC stress was expected to be different when different rebars were used. The effect of rebar stiffness on stress vs. strain relation is further elaborated in Section 2.5. It should be noted that the SHCC stress fluctuates once cracks are formed, so the stress level at the beginning of the long-term loading cannot be estimated precisely. The derived values based on the measured steel rebar strain are shown in Section 3.3.

The creep of the bridging rebar under the sustained loading was assumed to be negligible for both the steel and the GFRP. This is a reasonable assumption for steel. In the case of the GFRP, reference is made to a previous study [22] on the creep and relaxation behaviour of GFRP rebar. Results of this study indicate that the stress relaxation for helically wrapped GFRP rebar (which is the kind used in the present study) under an initial stress level of 20% of its tensile strength (which is the estimated GFRP stress in our experiment) is about 3.5% at 1000 h, and the test curve is already approaching a plateau. Up to about 56 days, which is the testing period in our study, the extrapolated relaxation was less than 4%. We consider this error to be acceptable in the current experiment. But the relaxation behaviour of the GFRP rebar can certainly be measured and included in future studies with the testing setup. In the creep test, the valve was closed upon quasistatically, reaching the target load for sustained loading, and the hydraulic pump was disconnected. The target loads were chosen such that the bridging rebar does not reach yield strength even upon the complete relaxation of the SHCC layer. As the applied load decreased due to the creep deformation of SHCC, the load was restored by moving the upper plate downwards using the topmost nuts on the thread bars. Over the duration of the test, the load was maintained within 1 kN of the target load. The test was conducted at 23 ± 2 °C and relative humidity 60% ± 10% and lasted at least 56 days. Nine SHCC specimens which had been cured for 14 days were placed under the same environment for the measurement of shrinkage strain according to ASTM C490/C490M [23]. The total strain measured during the creep stage was adjusted for shrinkage strain before the data were analysed.

To determine crack widths of the SHCC layer under sustained loading, images at specific locations of cracks were taken using a USB microscope (with a resolution of ~0.5 µm/pixel). Each region was demarcated with a line across the crack to identify the exact location of the image for future captures.

It should be noted that the strain of the steel bar may not have been zero at the beginning of the test (i.e., before the constant loading was quasistatically applied). This is because stresses can be induced in the rebar due to autogenous shrinkage of the SHCC during the curing stage or eccentricities introduced by the casting of an SHCC layer. A method to obtain the baseline strain value for an unstressed rebar is described at the end of the following section.

### 2.5. Variation in Stress–Strain during the Creep Test

**Figure 4:**
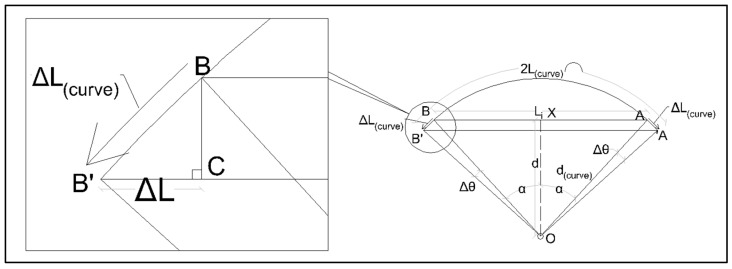
Elongation of an element attached to the concrete beam between two nodes AB at the centre.

To study the creep behaviour of SHCC under various conditions, it is desirable to have a means of estimating the stress–strain relation during the creep test, which reflects the degree of load redistribution between the SHCC and the reinforcing bar. This can be achieved through a simple geometric analysis. Assuming no debonding of the bridging rebar takes place inside the concrete of the half beams, the effect of an imposed tensile strain on a line element bridging the half beams is illustrated in Figure 4. The connection points will geometrically follow the path of an arc about the pivot.

Due to the symmetrical nature of the arrangement, only one side will be considered in the analysis.

An imposed strain of ε in the element results in the angle α being increased by Δθ due to the rotation.

(4) $$\Delta \theta =\frac{\Delta L_{curve}}{d_{curve}}=\frac{\left(\frac{\Delta L}{cos\alpha }\right)}{\left(\frac{d}{cos\alpha }\right)}=\frac{\Delta L}{d}=\frac{\varepsilon \times L_{i}/2}{d}$$

where $L_{i}$ refers to the initial linear length of the element between the two connection points and d refers to the perpendicular distance to the pivot. Similarly, in the case of SHCC and rebar load sharing, two elements can be considered as being rigidly connected at the nodes, with the angle Δθ being common to both. Therefore, the same relationship will follow for the element representing the steel rebar with d and $L_{i}$ being different.

Thereby, considering compatibility,

(5) $$\Delta \theta =\frac{\varepsilon_{rebar}\times L_{i,rebar}}{2\times d_{rebar}}=\frac{\varepsilon_{SHCC}\times L_{i,SHCC}}{2\times d_{SHCC}}$$

From moment balance,

(6) $$\Delta M=d_{rebar}\times F_{rebar}+d_{SHCC}\times F_{SHCC}$$

Using the material properties and substituting from Equation (5),

(7) $$\sigma_{SHCC}=\left[-{\left(\frac{d_{rebar}}{d_{SHCC}}\right)}^{2}\times \left(\frac{L_{i,SHCC}}{L_{i,rebar}}\right)\times \left(\frac{A_{rebar}}{A_{SHCC}}\right)\times E_{rebar}\right]\times \varepsilon_{SHCC}\;+\frac{M}{d_{SHCC}\times A_{SHCC}}$$

where *M* is kept constant for the duration of the creep test; hence, Equation (7) can be written as y = mx + c, where m is a negative number reflecting the change in stress with strain during the test. In the testing setup, $L_{i,SHCC}$ and $L_{i,rebar}$ are fixed. By changing the thickness/area of the SHCC layer and/or the rebar size and material (e.g., steel, GFRP), one can tune the value of m to produce different stress–strain paths. Although the simple geometric analysis is not exact, it is helpful in determining suitable parameters (such as steel rebar size and type) for the creep test.

**Figure 5:**
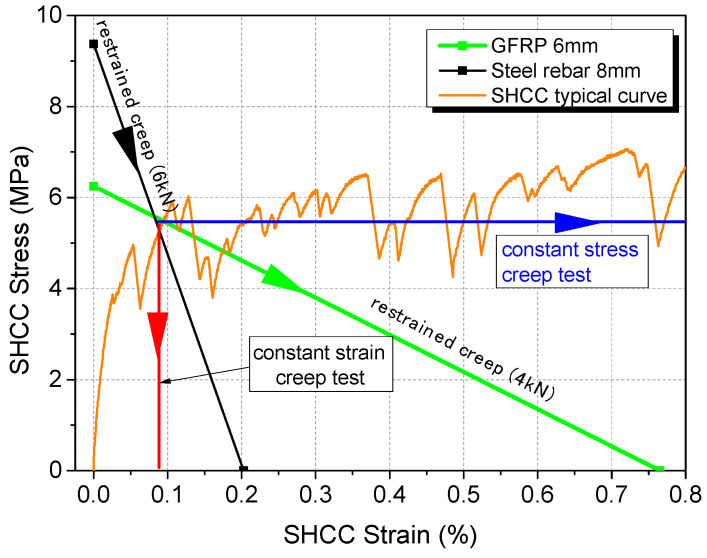
Stress–strain control of the restrained system.

For the SHCC layer employed in this study, the theoretical stress–strain relationship derived from the above equations for both steel rebar (8 mm) and GFRP rebar (6 mm) is illustrated in Figure 5, together with the constant stress and constant strain tests. Different from a constant strain or stress test, this system intrinsically dictates the path followed both by stress and strain with time, as indicated by the arrowhead. In this graph, the target load (before the creep test) can be obtained from a simple equilibrium calculation for a particular SHCC strain to be reached. In this study, different target loads (6 kN and 4 kN) were applied to the specimens with steel and GFRP rebar, respectively. With these loads, the initial equilibrium strain before creeping is expected to be similar for both cases, but the stress–strain imposed thereafter follows very different rates.

In the above derivation, *d_rebar_* and *d_SHCC_* are obtained from the distance between the centre of the hinge and the mid-heights of the rebar and SHCC layer, respectively. However, the hinge pin diameter was 25 mm. Considering the gap between the two half beams, there is a maximum uncertainty of ±10.5 mm as to the actual location of the pivot, depending on the stress distribution over the contact surface of the pin and steel block. To obtain a more accurate position of the pivot, the SHCC layer was cut after the creep test had been completed. Loading was applied again and since the applied moment was resisted by the rebar, the relationship between the imposed moment and rebar force (=rebar area × rebar stress derived from the strain gauge reading) was used to calculate the effective value of *d_rebar_* by taking *F_SHCC_* as zero in Equation (6). The rebar stress and SHCC stress presented in the results and discussions section were determined based on *d_rebar_*, obtained with this approach.

The baseline reading of the strain gauge reading (when the rebar in its unstressed state) was also established after the SHCC was cut and when there was no loading applied on the half beams. Under such conditions, the steel rebar should be unstressed. Knowing the baseline strain value, the absolute strain and stress in the rebar during the whole experiment was calculated and employed for deriving the SHCC stress.

## 3. Results and Discussion

### 3.1. SHCC Layer Loaded Quasistatically to Failure in the Fixture

**Figure 6:**
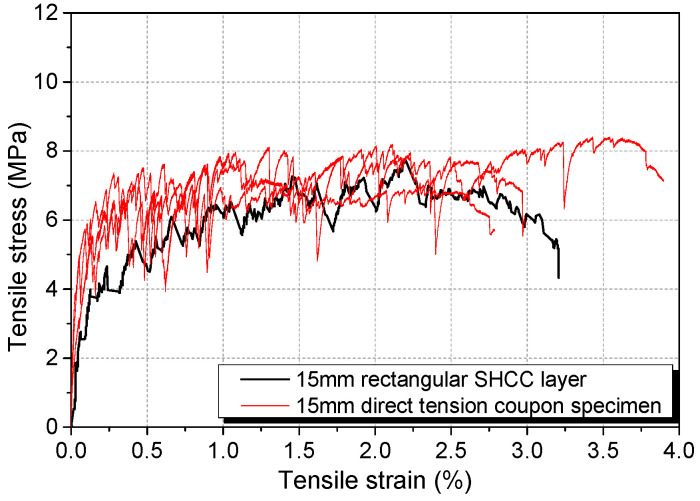
Comparison of stress–strain curves of SHCC layer with specimens tested in direct tension.

Figure 6 shows the stress–strain curve for an SHCC layer loaded quasistatically to failure in the fixture (Figure 2b), together with test results for the same mix composition from direct tensile tests performed on the UTM. Since it is not possible to calculate the exact pivotal distance, it was assumed to be the distance from the centre of the pin to the mid-height of the SHCC layer. With this assumption, the possible variation in SHCC stress is ±6%. With this uncertainty, the agreement between the results can be considered to be acceptable for the following reasons: (i) a similar stress–strain pattern with strain-hardening up to at least 2.0% of the strain (which was higher than the strain at the end of the creep test) was obtained from both cases; (ii) the difference between the two cases is within the typical variations of tensile behaviour between different batches of SHCCs with the same composition. Thus, despite the non-uniform distribution of stress across the depth of the SHCC layer and the curvilinear path followed by the end points, the fixture used in these proportions can be employed to study the direct tensile behaviour of SHCCs. Further verification of the similarity between tensile behaviour obtained from direct tensile tests and the fixtures will be discussed in Section 3.3.

An interesting observation can be made by closely inspecting the stress–strain behaviour for the two cases in Figure 6. When cracks were formed, almost vertical drops were observed in the SHCC specimens tested in the UTM, while the strain increased in the SHCC layer. This phenomenon is due to the direct tension test specimens being tested under displacement control, while the load system adopted for the fixture loading leans more towards force control, where strain can increase when a new crack is formed.

### 3.2. Calibration of Strain Gauges Attached to Bridging Rebar

**Figure 7:**
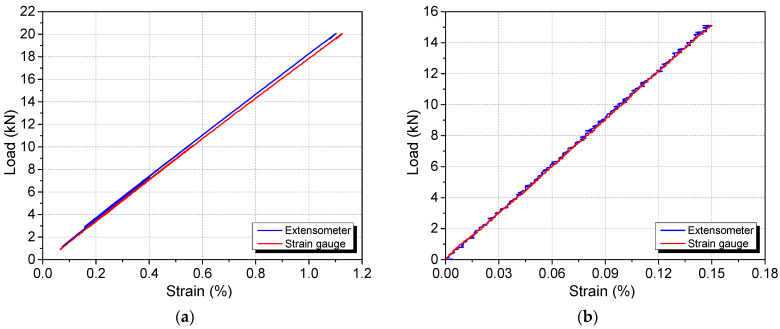
Tensile strain values based on the extensometer and strain gauges for (**a**) GFRP and (**b**) steel rebar.

The modulus of elasticity was determined to be 64 GPa and 205 GPa for the GFRP and high-yield steel bar, respectively, based on the extensometer readings, as shown in Figure 7. $A_{eff}$ was thereby determined to be 26.4 mm^2^ and 49.0 mm^2^ for the GFRP and steel rebar, respectively. The gradient of the load–strain line for the extensometer was predictably steeper, indicating that the location at which the strain gauges were attached experience a greater stress due to the area reduction. Linearity of the strain gauge was observed in the range of applied load. The strain limit of the strain gauge is 5%, which is well beyond the strain values in the high-yield steel and GFRP bar during our tests.

### 3.3. Application of Target Load and Monitoring Long-Term Stress–Strain Behaviour

**Figure 8:**
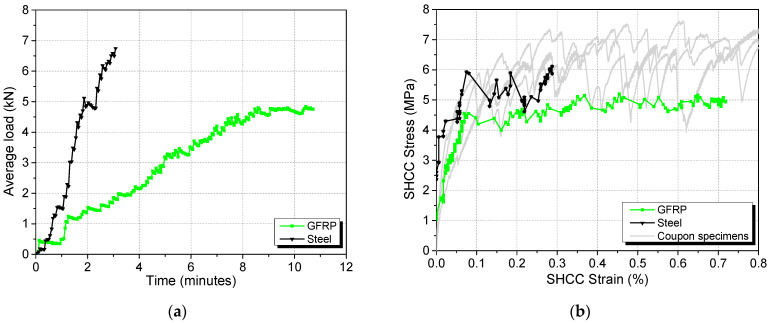
Development of (**a**) load and (**b**) stress in SHCC layer during the quasistatic loading phase.

Figure 8 shows the quasistatic loading phase before loading is fixed for creeping to occur. The system was loaded to the respective target loads within 10 min, as shown in Figure 8a, keeping load magnitudes at each end close to each other. The stress development of the SHCC layer during this loading phase is shown in Figure 8b. Also shown in the figure are the stress–strain curves obtained from direct tensile test (the same results as those shown in Figure 6). The agreement between the curves (within material variation) again verifies the applicability of the loading fixture in studying direct tensile behaviour.

**Figure 9:**
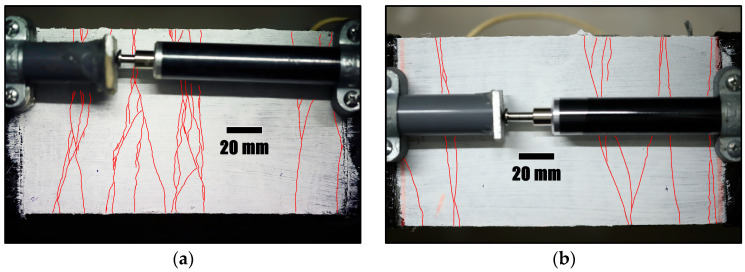
Surface cracking pattern (traced in red for clarity) at the end of the quasistatic loading phase in (**a**) GFRP and (**b**) steel specimen.

Henceforth, the specimens with GFRP and steel as the bridging rebars will be referred to as the GFRP specimen and the steel specimen, respectively. The tensile strain in the SHCC layer (derived from the LVDT readings) reached 0.72% and 0.29% in the GFRP and steel specimens, respectively, upon reaching the target load. More cracks were observed on the SHCC surface of the GFRP specimen than that of the steel specimen (see Figure 9).

**Figure 10:**
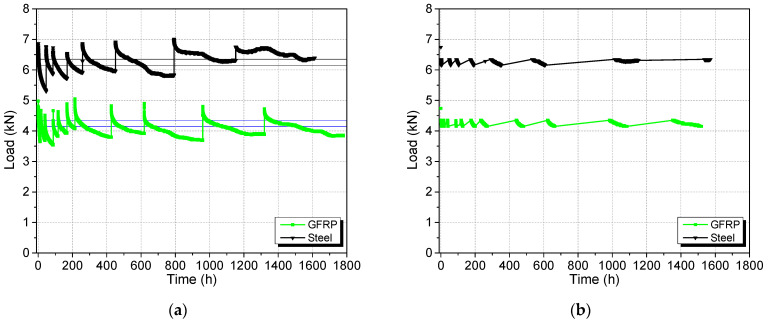
(**a**) Load–time (horizontal lines indicate the boundaries for filtering the dataset in each case) and (**b**) filtered load–time variation.

The load was kept within 1 kN of the target load throughout the sustained loading phase, as shown in Figure 10a. However, since the stresses in the rebar and SHCC are sensitive to small changes in the end loads, only the data points corresponding to the loads within 4.25 ± 0.1 kN and 6.25 ± 0.1 kN for the GFRP and steel specimen are used in the presentation of stresses in graphs, with gaps being linearly interpolated. This will allow the overall trend of the results to be clearly revealed. The bounds for the load envelope are depicted by the horizontal lines in Figure 10a and the subsequent filtered data points for loads are shown in Figure 10b.

**Figure 11:**
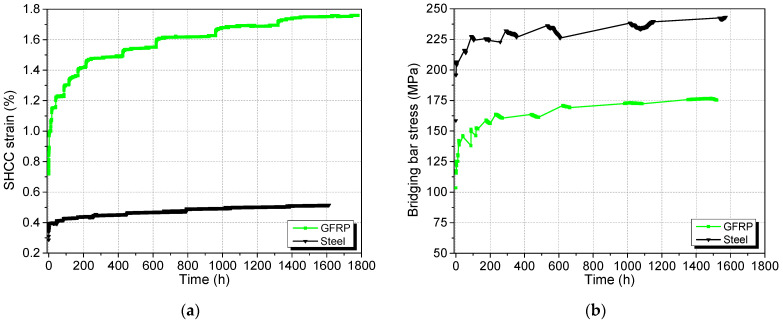
Variation in (**a**) SHCC strain and (**b**) stress in bridging rebar with time.

**Figure 12:**
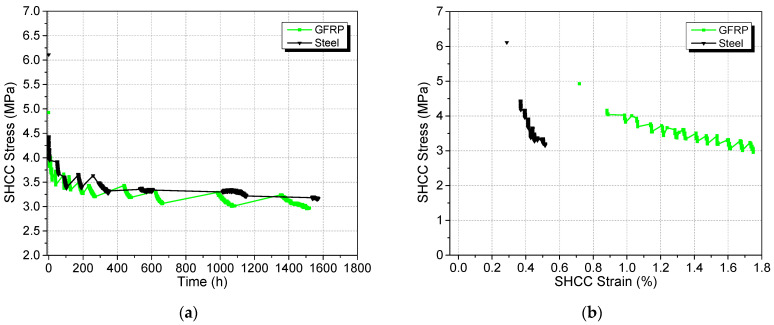
SHCC stress change with (**a**) time and (**b**) strain.

During the sustained loading phase of 56 days, the strain in the SHCC increased from 0.72% to 1.72% in the GFRP specimen and 0.29% to 0.51% in the steel specimen (see Figure 11a). Of the strain increase, 90% occurred within the first 500 h in both cases, and the rate of strain change decreased with time as the load carried by the SHCC layer was transferred to the rebar with increasing rotation. The increasing trend of the bridging bar stress with time observed in Figure 11b confirms the load being picked up by the rebar as the SHCC relaxes. The much larger increase in strain is associated with the lower elastic modulus of the GFRP compared to the steel rebar. Here, the GFRP bridging rebar has reached a load level of ~10% at 56 days. According to D’Antino and Pisani [22], relaxation up to 1000 h is only 2.2% and the curve is approaching a flat asymptote. Moreover, the measured stress vs. strain relation for the SHCC shows a linear correlation (see Figure 12b), as predicted by the geometric analysis. If the GFRP was creeping significantly, the measured strain in the GFRP would have exceed the elastic strain considerably, resulting in an underestimation of the calculated SHCC stress, and a nonlinear SHCC stress–strain relationship would be found. Based on these observations, we believe that it is acceptable to neglect the creep effect of GFRP in the experiment.

In Figure 11 and all subsequent figures showing SHCC strain, the effect of shrinkage has been removed. Measured shrinkage data which reached 0.05% at 56 days post-curing (a relatively low value in comparison to the strains recorded) was fitted to a power trendline. The time-dependent shrinkage value obtained from the fitted equation was then added to the strain derived from the strain gauge to eliminate the shrinkage strain from the total strain (which is tensile).

The first point shown in Figure 12a,b for each curve depicts the SHCC stress at the beginning of the sustained loading period. It should be noted that the loads corresponding to the first points do not fall within the filter range of 4.25 ± 0.1 kN and 6.25 ± 0.1 kN for the GFRP and steel specimen. These points are included to show compatibility between the quasistatic and sustained loading phases. The SHCC stress at the start of the creep test was 4.9 MPa and 6.1 MPa for the GFRP and steel specimens. The difference between the initial SHCC stresses in each case stems from the combination of the random nature of multiple cracking in SHCC, variability in the SHCC material and the SHCC strain at the end of the quasistatic loading phase. The tensile stress can also vary substantially while undergoing cracking, with sudden drops in stress corresponding to new crack formation, followed by increasing stress on further straining. Depending on when exactly a crack was formed, the SHCC stress at the end of quasistatic loading may correspond to a point just after the drop or right before a drop (i.e., before an additional crack was formed). The actual stress may therefore vary among different specimens.

The stress in the SHCC layer dipped below 4 MPa within 4 h of sustained loading in both cases albeit the SHCC layer of the steel specimen having a higher initial stress. Residual stress in the SHCC approached 3.2 MPa after 56 days in both cases. Note that this is just a coincidence. Higher loading was applied to the steel specimen, so the similarity of residual stress simply indicates that the steel rebar is carrying a higher share of the loading than the GFRP rebar, which is expected.

As shown in Figure 12b, a close linear correlation was observed between SHCC stress and strain for both the GFRP and the steel specimens (except for the first point which is outside the filter range). Although Equation (7) accurately predicted the linear nature of the relationship, the experimentally observed strain values were 240%–320% higher than the idealised strains from the geometric analysis. This could be due to the partial debonding of the part of rebar embedded in the half beam and other eccentricities induced previously. The gradient of the SHCC stress–strain line for steel specimen being higher than the GFRP specimen is consistent with the geometry analysis. Despite basing the target load in each case on having the same starting strain in the sustained-load phase, the SHCC layer on the GFRP specimen had about twice the strain that the SHCC layer on the steel specimen had.

As discussed above, a simple geometric analysis was performed to provide insight on the design of the test specimen, and it was not expected to predict the measured stress–strain relation accurately. Most importantly, the variations in the stress, strain, and crack development in SHCCs over time were all directly measured during the testing. Analysis of these data provide useful insights on the creep behaviour of SHCCs.

### 3.4. Development of Crack Width

**Figure 13:**
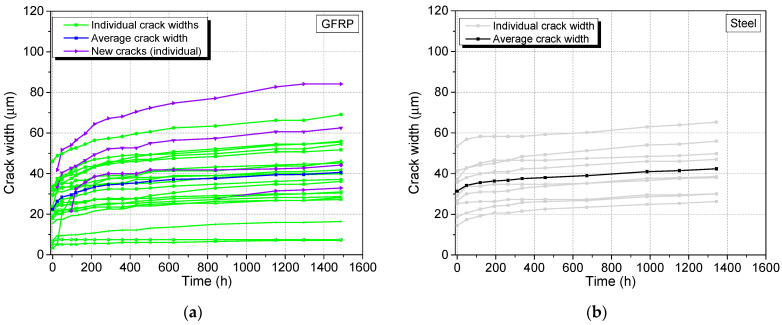
Individual and average crack width development of the SHCC layer in the (**a**) GFRP and (**b**) steel specimens with time.

**Figure 14:**
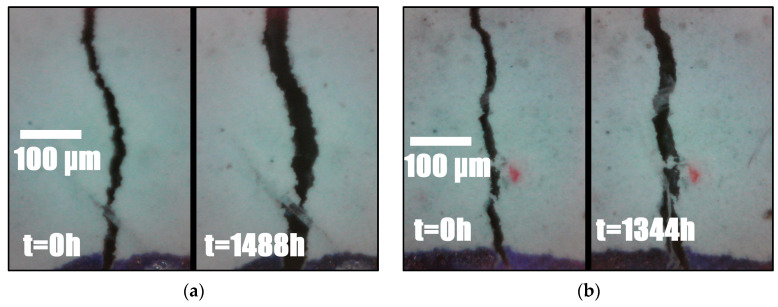
Initial and final image for a crack in the SHCC layer of (**a**) GFRP and (**b**) steel specimen.

At the end of the quasistatic loading phase, a total of 20 cracks and 9 cracks were present on the GFRP and steel specimens. The cracks in the GFRP and steel specimens appeared to be widening simultaneously during the sustained loading period, as shown in Figure 13. Although new cracks did not appear on the steel specimen after the initial quasistatic loading phase, new cracks were observed in the GFRP specimen on days 1, 4 and 35. As both specimens had similar stress values during relaxation, this can be due to the higher strain rate imposed on the GFRP specimen, which is closer (but not identical) to the situation of constant stress in comparison. On the other hand, no new cracks formed in the steel specimen, which is closer to the constant strain situation. The image of a crack as seen by the microscope is shown in Figure 14.

Notably, none of the cracks surpassed 100 µm at the 56-day mark, while the average crack width stayed under 50 µm. At 56 days, however, the crack width–time curves generally did not reach a plateau in either of the specimens. With the developed load-sharing fixture, tests with longer durations can be carried out in the future.

**Figure 15:**
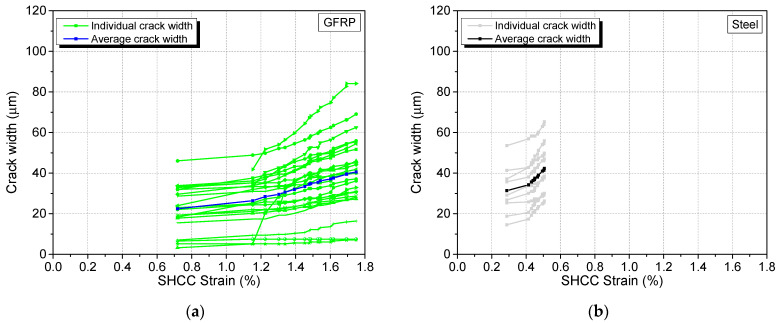
Individual and average crack width development of the SHCC layer in the (**a**) GFRP and (**b**) steel specimens with strain.

As observed in Figure 15, aside from the initial jump in the strain (indicated by the almost horizontal portion) which occurred during the first two days, the average crack width shows a mostly linear trend with strain in both the GFRP and steel specimens. In the absence of new cracks, the strain increase is being accommodated for in part by the expansion of the existing crack widths. The lower initial strain and lower number of cracks present in the steel specimen results in a steeper average crack width–strain curve as compared to the GFRP specimen.

**Figure 16:**
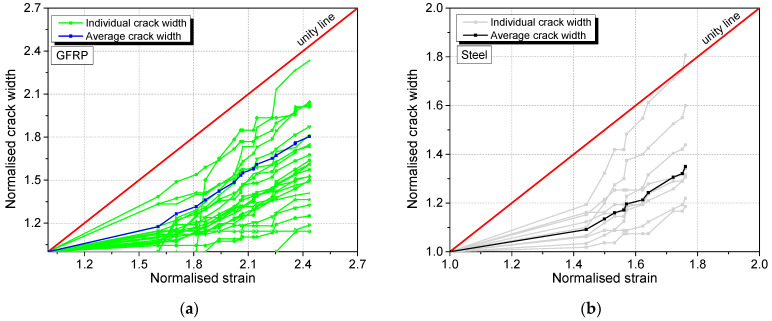
Normalised individual and average crack width development of the SHCC layer in the (**a**) GFRP and (**b**) steel specimens with normalised strain.

Considering the normalised crack width development with the normalised strain shown in Figure 16, the normalised average crack width develops below the line of unity, implying that the overall crack widening is less pronounced than the increasing of strain. For the GFRP specimen, this observation can be partially explained by the formation of new cracks so the average opening of each crack at a given strain is decreasing. However, no new cracks were formed in the steel specimen. Also, for both specimens, the sum of the measured crack openings was well below the product of the total strain and the gauge length. Since the elastic strain in the material between the cracks was very small and could not make up the difference, there must be a nonlinear deformation in the matrix that has not been accounted for. This may include matrix creeping and fine microscopic damages that are not detectable by the microscope. Future investigations on these aspects are warranted.

An outlier has been excluded from Figure 16a (but was still included in the average calculations) due to a crack expanding from 3 µm to 46 µm (which is still a small value). The normalised crack width in this case shows an abnormally large jump in the dataset.

As opposed to crack localisations occurring and the failure of SHCC within few days in constant stress tests [15], the SHCC in both the GFRP and steel specimens showed no cracks widening exponentially with time during the testing period.

The crack analysis based on the test results from the load-sharing fixture provides important insights on the creep behaviour of SHCCs in practical situations, where loading redistribution enables the loading to decrease in the SHCC. Under such a condition, the rapid increase in crack opening (which may even lead to creep rupture) in SHCCs under constant loading does not occur. In addition, the increase in normalised crack width (i.e., current crack width/crack width when creeping starts) is found to be below the increase in normalised strain (current strain/strain when creeping starts). This can provide a preliminary guideline for the durability design of SHCC members. Providing the crack width vs. strain relation of the SHCC has been established from direct tensile testing; the strain can be calculated for the serviceability load acting on the structural member to obtain the crack width (based on crack width vs. strain relation measured from a tensile test). Creep analysis (to be discussed in the following section) can then be performed to find the change of strain in the member with time. A conservative estimate of the crack width after creeping can be taken as the product of the initial crack width and the ratio of the strain before and after creeping. With the load-sharing system, additional experiments will be carried out with specimens containing different reinforcing bars to further verify/support the proposed design approach with test results for different stress–strain relations during creep.

### 3.5. Deriving Creep Compliance for the SHCC

For an RC member with an SHCC layer at the tensile surface (or an SHCC member with a rebar), the deformation due to creep can be derived if the creep behaviour for the SHCC and the concrete are known. Under the applied loading, the creep strain in the SHCC and concrete can be calculated separately first. To maintain compatibility, the SHCC stress will have to decrease while stress in the steel would increase. Through an iterative process, the final distribution of stress and strain in a particular section (which is under a certain applied moment) at a given time can be derived. Knowing the strain increase, the increase in crack width can be estimated, as discussed above.

The creep behaviour of concrete can be found in design codes. For the SHCC, one can measure the creep compliance J(t), which is the deformation per unit stress at a given time after a constant stress is applied. From its definition, the most direct way to obtain J(t) is through a constant loading creep test. However, as discussed above, constant loading will lead to excessive crack opening and premature rupture failure that is not found in more practical situations. The creep compliance obtained under such a loading condition is therefore also likely to severely overestimate the creep strain in practice. Here, an approach to derive J(t) from the measured stress and strain values obtained in the load sharing creep test is proposed.

When loading is shared between the SHCC layer and the steel rebar, the SHCC stress is no longer constant. Under varying stress, σ(t), the strain ε(t) at any time, t, can be derived from a convolution integral:

(8) $$\varepsilon (t)=\sigma (0)J(t)+\int_{0}^{t}J(t-\tau )\;\frac{d\sigma \left(\tau \right)}{d\left(\tau \right)}\;d\tau$$

To analyse the results for the SHCC, we note that the starting point for the creep test is already in the hardening regime of the material. Focusing on the further increase in strain due to creep, the inelastic strain at the starting point is taken out so the strain at *t* = 0 (end of quasistatic loading or beginning of the creep regime) is taken to be $\varepsilon \left(0\right)=\sigma \left(0\right)/E$, with $\sigma \left(0\right)$ being the stress when creep starts and E being the elastic modulus of the SHCC. J(0) (or $J_{0}$) is then given by 1/E.

To find J(t), the total testing time is first divided into N equal intervals, $t^{\prime }$, with $t_{n}=nt^{\prime }$. J(t) is then taken to be a multilinear function with values of $J_{1},\;J_{2},\;..,J_{n}$ at $t_{1},t_{2},..,t_{n}$, respectively. Similarly, the stress and strain are taken to vary linearly within each time interval and their values at time t_n_ are taken to be $\sigma (t_{n})$ and $\varepsilon (t_{n})$, respectively. The integral in Equation (8) can then be turned into a summation over time intervals up to the time of interest. Within a time interval between $t_{n-1}$ and $t_{n}$, dσ(τ)/dt is a constant equal to $\left[\sigma \left(t_{n}\right)-\sigma \left(t_{n-1}\right)\right]/t^{\prime }$ and the integral of $J_{t-\tau }$ is $t^{\prime }$ multiplied by the average between $J_{n-1}$ and $J_{n}$ at the starting and end points of the interval, respectively. The following relations can then be derived from algebra.

(9) $$\varepsilon \left(t_{1}\right)=\sigma \left(0\right)\left(J_{1}-J_{0}\right)/2+\sigma (t_{1})(J_{1}+J_{0})/2$$

For *n* > 1

(10) $$\varepsilon \left(t_{n}\right)=\sigma (0)(J_{n}-J_{n-1})/2+\sum_{i=1}^{n-1}[\sigma \left(t_{n-i}\right)(J_{i+1}-J_{i-1}/2)]+\sigma (t_{n})(J_{1}+J_{0})/2\;$$

From Equation (9), $J_{1}$ can be obtained. Then, based on continuous application of (10), $J_{2},\;J_{3},\;..,J_{n}$ can be derived sequentially.

With more test results generated from the proposed load-sharing system, J(t) will be numerically derived for various initial strains (i.e., strain when creep starts) and stress vs. strain relation during creep. Ideally, the creep compliance function is the same (within experimental error) for all cases. If not, more detailed investigation will be carried out to understand the mechanisms behind the difference and how they can be considered in practical design.

## 4. Conclusions

In this study, a novel load-sharing system was developed to study tensile creep of SHCCs in practical situations where loading can be redistributed to the reinforcements in a structural member. The system was set up to capture the SHCC stress, strain, and crack width changes with time. In the test, the SHCC stress and strain are both intrinsically controlled by the system, with the imposed stress–strain being governed by the member dimensions, reinforcement stiffness and area, and applied load. Example test results from the system were presented to illustrate the new insights that can be obtained. The main findings of the study are summarised below.

1. For a fixture with the proportions used in this study, the tensile behaviour measured on an SHCC layer was similar to that obtained from the direct tensile test. Reasonable agreement was achieved up to at least 2.0% tensile strain, which is beyond the range for the studying of creep behaviour under practical conditions.
2. The fixture was able to impose a specific self-controlled stress–strain regime on the SHCC layer, with the surface of the SHCC being accessible for visual inspection and displacement instrumentation. It was also possible to derive the stress in the SHCC for the duration of the test by using strain gauges attached to the rebar with epoxy and multilayer protection.
3. A linear correlation was observed between SHCC stress and strain during the sustained loading phase as expected from the load-sharing system based on a simple geometric analysis. While the geometric analysis cannot predict the stress and strain values accurately, it is useful in forming a basis for the selection of relevant parameters (e.g., reinforcement material and size) in the test.
4. With the SHCC in the GFRP and steel specimen showing a 240% and 160% increase in strain, respectively, during the sustained loading phase, all cracks remained below 100 µm and the average crack width remained below 50 µm during the sustained loading phase. New cracks were only formed in the GFRP specimen, owing to its higher strain rate. This implies that the probability of forming new cracks to accommodate strain increase becomes lower when the imposed stress–strain tends more towards constant strain regime, such as the situation with stiff reinforcements restraining creep deformation.
5. The drastic increase in crack opening and premature failure for SHCC reported in the literature for constant creep test may not occur for practical conditions when there is load sharing between the SHCC and the reinforcements in the member.
6. The average crack width increase that occurred in the SHCC due to creep was not as pronounced as the increase in strain. As a preliminary design approach, one can conservatively estimate the crack width after creep as the product of the crack width before creep and the ratio of strain values before and after creep.
7. With this setup and instrumentation, the variation in stress and strain with time can be measured. An approach is proposed to derive the creep compliance of SHCC from the data. The obtained compliance function should be more applicable to real situations than that from the constant-stress creep test, which tends to induce excessive crack opening and creep deformation.

## Data Availability

Data are contained within the article.

## References

[1] Li V.C. (2003). On Engineered Cementitious Composites (ECC). J. Adv. Concr. Technol..

[2] Li V.C. (2008). Engineered Cementitious Composite (ECC): Material, Structural, and Durability Performance.

[3] Li V., Wang S., Wu C. (2001). Tensile strain-hardening behavior of polyvinyl alcohol engineered cementitious composite (PVA-ECC). Mater. J..

[4] Ranade R., Li V.C., Stults M.D. (2013). Composite Properties of High-Strength, High-Ductility Concrete. ACI Mater. J..

[5] Kamal A., Kunieda M., Ueda N., Nakamura H. (2008). Evaluation of crack opening performance of a repair material with strain hardening behavior. Cem. Concr. Compos..

[6] Wang K., Jansen D.C., Shah S.P., Karr A.F. (1999). Permeability Study of Cracked Concrete. Mater. Struct..

[7] Lepech M.D., Li V.C. (2009). Application of ECC for bridge deck link slabs. Mater. Struct..

[8] Li V.C. (1997). Engineered Cementitious Composites (ECC)—Tailored Composites Through Micromechanical Modeling. Fibre Reinforced Concrete: Present and the Future.

[9] Djerbi A., Bonnet S., Khelidj A., Baroghel-bouny V. (2008). Influence of traversing crack on chloride diffusion into concrete. Cem. Concr. Res..

[10] Yu J., Li H., Leung C.K.Y., Lin X., Lam J.Y.K., Sham I.M.L., Shih K. (2017). Matrix design for waterproof Engineered Cementitious Composites (ECCs). Constr. Build. Mater..

[11] (2013). Code of Practice for Structural Use of Concrete.

[12] Maalej M., Li V.C. (1995). Introduction of Strain-Hardening Engineered Cementitious Composites in Design of Reinforced Concrete Flexural Members for Improved Durability. ACI Struct. J..

[13] Leung C.K.Y., Cao Q. (2010). Development of pseudo-ductile permanent formwork for durable concrete structures. Mater. Struct..

[14] Boshoff W.P., Mechtcherine V., van Zijl G.P.A.G. (2009). Characterising the time-dependant behaviour on the single fibre level of SHCC: Part 1: Mechanism of fibre pull-out creep. Cem. Concr. Res..

[15] Boshoff W.P., Adendorff C.J. (2013). Effect of sustained tensile loading on SHCC crack widths. Cem. Concr. Compos..

[16] Boshoff W.P. (2014). Cracking Behavior of Strain-Hardening Cement-Based Composites Subjected to Sustained Tensile Loading. ACI Mater. J..

[17] Ratnayake K.A.S.D., Leung C.K.Y. Cracking behaviour under creep in strain-hardening cementitious composites (SHCC) applied as a protective layer on reinforced concrete flexural elements. Proceedings of the 11th International Conference on Fracture Mechanics of Concrete and Concrete Structures.

[18] Ratnayake K.A.S.D., Li K.W., Leung C.K.Y. (2023). Cracking Behaviour of Strain-Hardening Cementitious Composites (SHCC) Under Practical Creep Conditions. Proceedings of the Strain Hardening Cementitious Composites SHCC5.

[19] Chen Y., Yu J., Leung C.K.Y. (2018). Use of high strength Strain-Hardening Cementitious Composites for flexural repair of concrete structures with significant steel corrosion. Constr. Build. Mater..

[20] Coover H.W., Dreifus D.W., O’Connor J.T., Skeist I. (1990). Cyanoacrylate Adhesives. Handbook of Adhesives.

[21] Millet G.H., Hartshorn S.R. (1986). Cyanoacrylate Adhesives. Structural Adhesives: Chemistry and Technology.

[22] D’Antino T., Pisani M.A. (2019). Long-term behavior of GFRP reinforcing bars. Compos. Struct..

[23] (2021). Standard Practice for Use of Apparatus for the Determination of Length Change of Hardened Cement Paste, Mortar, and Concrete.

